# Evaluating a couple communication skills training (CCST) intervention for advanced cancer: study protocol for a randomized controlled trial

**DOI:** 10.1186/s13063-022-06656-4

**Published:** 2022-08-26

**Authors:** Laura S. Porter, Katherine Ramos, Donald H. Baucom, Karen Steinhauser, Alaattin Erkanli, Timothy J. Strauman, S. Yousuf Zafar, Devon K. Check, Karena Leo, Evan Liu, Francis J. Keefe

**Affiliations:** 1 Department of Psychiatry & Behavioral Sciences, Duke University School of Medicine, DUMC 102506, Durham, NC 27710 USA; 2 Department of Psychology, University of North Carolina at Chapel Hill, Chapel Hill, NC USA; 3 Population Health Sciences Department of Medicine, Duke University School of Medicine, Durham, NC USA; 4 Department of Biostatistics and Bioinformatics, Duke University School of Medicine, Durham, NC USA; 5 Department of Psychology, Duke University, Durham, NC USA; 6 Department of Medicine, Duke University School of Medicine, Durham, NC USA; 7 Population Health Sciences, Duke University School of Medicine, Durham, NC USA; 8 Trinity College of Arts & Sciences, Duke University, Durham, NC USA

**Keywords:** Cancer, Communication, Videoconferencing, Caregivers, Randomized controlled trial

## Abstract

**Background:**

For patients and their intimate partners, advanced cancer poses significant challenges that can negatively impact both individuals and their relationship. Prior studies have found evidence that couple-based communication skills interventions can to be beneficial for patients and partners. However, these studies have been limited by reliance on in-person treatment delivery and have not targeted couples at high risk for poor outcomes. This study tests the efficacy of a Couples Communication Skills Training (CCST) intervention delivered via videoconference for couples reporting high levels of holding back from discussing cancer-related concerns, a variable associated with poorer psychological and relationship functioning.

**Methods:**

This RCT is designed to evaluate the efficacy of CCST in improving patient and partner relationship functioning (primary outcome). Secondary outcomes include patient and partner psychological functioning and patient symptoms and health care use. We also examine the role of objective and self-reported communication behaviors as mediators of treatment effects. Two hundred thirty patients with advanced lung, gastrointestinal, genitourinary, and breast cancer and their partners will be randomized to CCST or an education control intervention. Participants in both conditions complete self-reported outcome measures at baseline, mid-treatment, post-treatment, and 3 months post-treatment. Objective measures of communication are derived from video-recorded couple conversations collected at baseline and post-treatment. An implementation-related process evaluation (assessing implementation outcomes and potential barriers to/facilitators of implementation) will be conducted to inform future efforts to implement CCST in real-world settings.

**Discussion:**

This trial can yield important new knowledge about effective ways to improve patient and partner adjustment to advanced cancer.

**Trial registration:**

This study trial is registered at clinicaltrials.gov (Trial # NCT04590885); registration date: October 19, 2020.

**Supplementary Information:**

The online version contains supplementary material available at 10.1186/s13063-022-06656-4.

## Introduction

For patients and their intimate partners, advanced cancer poses significant challenges that can negatively impact them individually and as a couple. Patients typically undergo multiple treatments and experience a variety of disease and treatment-related symptoms including fatigue, pain, sexual problems, and sleep difficulties [1–4]. These symptoms can limit patients’ ability to perform usual family and workplace responsibilities, disrupting their role functioning in important areas [5, 6]. Many patients also experience psychological distress, including depression and anxiety [7, 8], fears of disease progression and death [9, 10], ,and feelings of hopelessness, guilt, and loss of meaning [11].

Advanced cancer likewise poses formidable challenges for caregiving partners and patient-partner relationships [12–14]. Partners attempt to provide emotional and physical support for the patient and cope with their own psychological difficulties, anticipatory grief, and existential distress [15, 16]. Disruptions that patients experience in family and work roles affect their partners who often take over tasks the patient can no longer perform [5, 13]. These caregiving demands can create an imbalance in the relationship that may lead to burnout, relationship dissatisfaction, and caregiver strain [17].

Couples’ ability to communicate openly and effectively with each other about cancer-related concerns can improve their psychological adjustment and relationship quality [18]. It may also lead to better symptom management for the patient and medical decisions that align with the patient’s goals and priorities. However, many couples report difficulties communicating about cancer, even in the context of overall satisfying relationships [19]. This can lead to deficits in support, decreases in intimacy and relationship quality, and increased psychological distress. Thus, interventions designed to facilitate effective patient-partner communication are likely to have beneficial effects for both individuals and their relationship.

Prior research, including studies conducted by our team, indicates that couple-based interventions that target communication lead to positive outcomes [20–22]. However, most studies have been limited by reliance on in-person treatment delivery formats, precluding many couples from participating due to barriers such as the patient’s symptoms, distance, and time. Also, prior studies have not targeted interventions to couples most likely to need and benefit from treatment, i.e., those at risk of poor outcomes [21, 23]. Our prior research indicates that couples who report communication difficulties (e.g., high levels of holding back from discussing cancer-related concerns) have increased psychological distress and poorer relationship functioning and are most likely to benefit from a couple communication intervention [21, 24].

We are thus conducting a randomized clinical trial (RCT) to assess the efficacy of a couple communication skills training (CCST) intervention delivered via videoconference for couples facing advanced cancer, targeted to couples who report high holding back from discussing cancer-related concerns. The CCST intervention includes components to help couples communicate effectively, decrease avoidance of important cancer-related issues, and provide each other with support. The study also systematically evaluates objective and self-reported indices of communication as mediators of treatment effects. This project builds upon a pilot study which demonstrated the feasibility and acceptability of this approach [22]. Here we present the study design, intervention, outcomes, and analysis plan for the RCT.

## Methods

### Study design

**Fig. 1:**
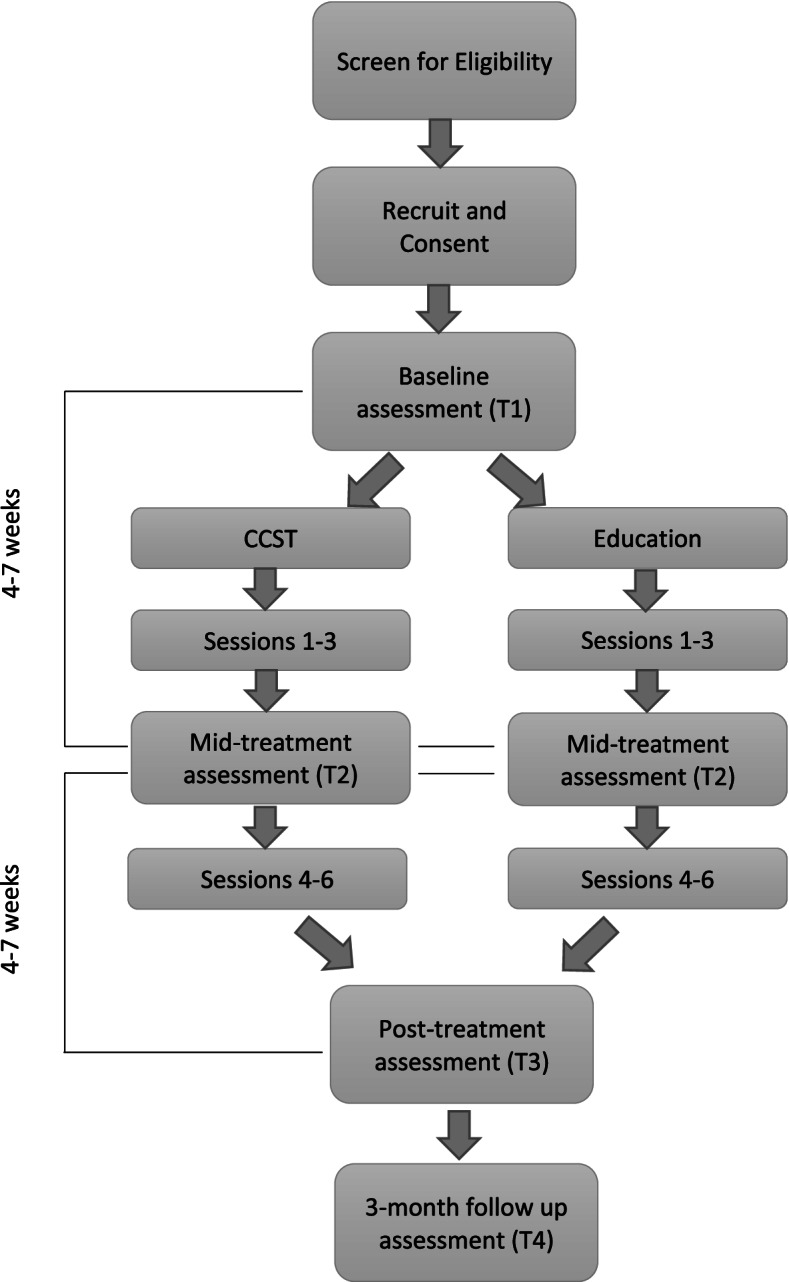
Study flow

This is a two-armed RCT in which 230 patient-partner dyads are randomized to (a) Couple Communication Skills Training (CCST) or (b) an education control condition. The study flow is shown in Fig. 1. In both arms, dyads participate in six weekly videoconference sessions with a trained interventionist. The primary endpoint is patient and partner relationship functioning. Our aims are as follows: (1) test the hypothesis that the CCST intervention will significantly improve patients’ and partners’ relationship functioning (i.e., intimacy and relationship satisfaction) compared to the education control intervention; (2) test the hypothesis that CCST significantly improves patients’ and partners’ individual psychological adjustment (i.e., psychological distress, affect, life completion) and patient health and health care outcomes (physical well-being, symptom distress, advance care planning discussions and completion of advance directives, hospitalizations, and emergency department visits) compared to the education intervention; and (3) test the hypothesis that, for couples receiving the CCST intervention, improvements in psychological adjustment, relationship functioning, and patient health are mediated by improvements in their communication, including self-reported protective buffering and objective measures of communication quality and communal coping (e.g., “we-talk”) derived from couple conversations.

There are two exploratory aims (1): to examine differences in response to the CCST intervention for patients versus partners, for male versus female participants, and for patients with different cancer diagnoses and (2) to conduct an implementation-related process evaluation of the intervention.

The SPIRIT guidelines [25] have been followed for this protocol, and the schedule of enrollment, interventions, and assessments is shown in Table 1. The SPIRIT Checklist is provided as an additional file (see Additional file 1). All items from the current registry can be found within this protocol. Patient recruitment and data collection began in October, 2020.

**Table 1:** Schedule of enrollment, interventions, and assessments

	Enrollment	Allocation	Post-allocation			
	Eligibility	Baseline	Sessions	Mid-treatment	End of treatment	3 months
	T0	T1	1-6	T2 ^a^	T3 ^b^	T4 ^c^
**Enrollment**						
Eligibility screen	X					
Informed consent		X				
Allocation		X				
**Interventions**						
CCST Intervention			X			
Education Intervention			X			
**Measures**						
**Primary outcomes**						
Miller Social Intimacy Scale (MSIS)		X		X	X	X
Couple Satisfaction Index-8 (CSI-8)		X		X	X	X
**Secondary outcomes**						
Hospital Anxiety and Depression Scale (HADS)		X		X	X	X
Positive and Negative Affective Scale (PANAS)		X		X	X	X
Life Completion subscale of the Quality of Life at the End of Life (QUAL-E)		X		X	X	X
Physical Well-being subscale of the Functional Assessment of Cancer Therapy (FACT)^d^		X		X	X	X
Physical Symptom subscale of the Condensed Memorial Symptom Assessment Scale (CMSAS)^d^		X		X	X	X
Patient health care use, advance care planning discussions, and advance directives^d^		X		X	X	X
Treatment satisfaction					X	
**Mediators**						
Self-reported communication (Protective Buffering Scale, PBS)		X		X	X	X
Couple conversations coded for objective indices of communication: Linguistic Inquiry and Word Count (LIWC), Asymmetric Behavior Coding System (ABCS), Relational Affective Topography System (RATS)		X			X	

^a^T2 occurs approximately 4 weeks following T1

^b^T3 occurs approximately 7 weeks following T1

^c^T4 occurs approximately 13 weeks following T1

^d^Patient-only measure

### Setting and eligibility criteria

This study is being conducted at Duke Cancer Institute (DCI) located in Durham, North Carolina. DCI is an NCI designated Comprehensive Cancer Center that treats the majority of patients with cancer from the local community and serves as a regional referral center, with more than 175,000 outpatient encounters and more than 7,000 new cancer patients seen annually. Approval was obtained from the Duke University Health System Institutional Review Board (#Pro00103232).

Patient inclusion criteria include the following: (a) diagnosis of stage IIIB-C or stage IV non-small cell lung cancer or extensive stage small cell lung cancer, stage III pancreatic cancer, or stage IV gastrointestinal (GI), genitourinary (GU), or breast cancer; and (b) married or in a committed intimate relationship. Patients and partners must speak and read English and be ≥ age 18. Exclusion criteria for patients and partners include the following: (a) lack capacity for interview (documented diagnosis of active psychosis or dementia); (b) physical impairment that precludes the use of a computer or videoconferencing; or (c) too sick to participate, as judged by the oncologist or research staff. There are no restrictions on concomitant care received during the trial.

As we are interested in the subset of couples who are experiencing difficulty talking about cancer-related concerns, we screen potential participants using a shortened version of the Holding Back subscale of the Emotional Disclosure Scale [24, 26]. The original subscale measures active inhibition of expression of cancer-related concerns to one’s partner in ten domains. Reported means from studies with patients with GI, GU, breast, and lung cancer have ranged from 0.82 (SD = 0.99) [27] to 1.23 (SD = 1.03) [24]. Higher scores on this scale have been consistently associated with increased psychological distress and poorer relationship functioning [24, 26, 28, 29]. To decrease participant burden, we created a shortened version of the scale. Using data from a prior study of 236 cancer patient-partner dyads, we identified the five items that correlated most highly with the total score which we used in the shortened version. Scores on the 5-item subscale correlated 0.93 with the 10-item scores. The 5-item subscale demonstrated good internal consistency (Cronbach alpha = 0.85) and correlations with measures of relationship satisfaction and psychological distress that were nearly identical to those found with the original measure. In the current study, the study coordinator administers the 5-item Holding Back screen to patients and partners separately over the telephone; as in our pilot study, couples in which one or both partners scores ≥1.0 are invited to participate.

### Recruitment and enrollment

All recruitment procedures comply with Health Insurance Portability and Accountability Act (HIPAA) guidelines. Potentially eligible patients are identified from oncology providers’ clinic schedules, institution tumor registries, or clinician referral. Introductory study letters are sent to preliminarily eligible patients. Patients who do not decline further contact are contacted by a research coordinator who provides information about the study, screens the patient and partner for eligibility, and reviews study procedures with the couple. The research coordinator reviews the consent form with eligible, interested participants and obtains informed consent using web-based forms (or paper consent forms if necessary).

### Randomization and blinding

Following patient and partner completion of informed consent and the baseline assessment, dyads are randomized with equal allocation to one of two treatment conditions: (a) Couples Communication Skills Training (CCST) or (b) an education control condition. Randomization is stratified by patient sex and occurs in blocks of four, which guarantees that after every four assignments the study arms will have equal numbers. Randomization is performed using Research Electronic Data Capture (REDCap), a secure web-based tracking and online data acquisition system. At the start of the study, the statistician generated random allocation tables which were uploaded into REDCap. After a dyad completes the baseline assessment and the interventionist schedules the first treatment session, the research coordinator prompts REDCap to check the allocation table and display the group to which the dyad is randomly assigned. This assignment is permanent and not editable within the participant record nor modifiable in the audit log. Study personnel and participants remain blind to the treatment arm to which they are assigned until after the baseline assessment is complete and the first treatment session is scheduled. Study personnel involved in collecting data post-allocation are blinded throughout the study.

### Intervention arms

#### Couples Communication Skills Training (CCST)

The CCST protocol is delivered to patient-partner dyads in six, 60-minute sessions conducted by a therapist via videoconference. The sessions are intended to be delivered at weekly intervals. However, realizing that patient and partner health or other circumstances may arise that can delay sessions, we will allow for the 6 sessions to be completed within 12 weeks (from the start of the first session) and will consider completion of all 6 sessions within 14 weeks of randomization to be within protocol. Couples who do not have access to a device for videoconferencing are loaned a tablet computer (iPad) with internet access for use during the intervention phase of the study.

The CCST intervention is informed by general principles of healthy relationship functioning and includes components to assist couples in communicating effectively, provide each other with support, and maintain some semblance of normality in their lives [18, 30]. It includes training in communication skills for (a) sharing one’s thoughts and feelings (e.g., disclosure) and listening to one’s partner and responding in a supportive manner and (b) joint problem solving. Couples’ avoidance in addressing cancer-related discussions is addressed through assessing their motivations for avoidance, validating and normalizing the couple’s concerns, and describing the potential benefits of addressing difficult topics [30]. Training includes both didactic and experiential components and is summarized in handouts. We individualize the skills training by incorporating information gathered from the couple about their communication style and challenges, focusing on the components that are the most relevant and helpful for them, and eliciting their feedback throughout the sessions. At the end of each session, the therapist instructs the couple to practice using the communication skills at home. Home practice is reviewed at the beginning of each session.

The first session teaches couples communication skills for disclosing thoughts and feelings about cancer, along with communication strategies for accepting and affirming the other person’s feelings and perspectives [31]. The second session focuses on training couples in skills for joint problem-solving, an intervention that has been demonstrated to be efficacious with couples in a variety of contexts [32]. In sessions 3–6, the therapist presents a brief review of the communication skills. The couple then practices using the skills during conversations, with the therapist providing feedback. We provide couples with a list of possible topics to discuss which cover a range of issues related to the cancer experience (Table 2). During sessions 1–3, the therapist encourage couples to talk about any cancer-related issue that is of personal concern. In sessions 4–6, the therapist guides couples to discuss topics that they may still be avoiding and/or those that may facilitate transitions at end of life. The last session also includes discussions of the couple’s progress during treatment and future issues the couple anticipates addressing relative to communicating about cancer.

**Table 2:** CCST topic list

**Health experience** • Your reaction to the diagnosis • Managing symptoms such as pain and fatigue • Dealing with changes in your physical appearance • Stigma of having a cancer diagnosis • Conflict with health care providers • Treatment decisions, including goals of care and advance care planning	**Reflection** • Moments of your life that were most important to you • Things you might have done differently • Things you would still like to accomplish • Things you would like to share with future generations (what do you want your grandchildren to know about you?) • Fears or worries about dying • Plans for the future
**Practical issues** • Financial difficulties • Having to give up or cut back from work or other important activities • Difficulties completing daily activities and household tasks • Disruptions to your life caused by cancer	**Family and friends** • Telling family members, friends, or co-workers about your illness • Caring for your children • Maintaining relationships with friends and family • Maintaining physical intimacy with your partner

#### Education intervention

Couples in this condition also receive six sessions delivered via videoconference. The therapist and the scheduling of the sessions is the same as for the CCST intervention. The education intervention provides couples with health information relevant to cancer in a supportive environment. Sessions focus on the following topics: fatigue, sleep disturbance, nutrition, physical activity, survivorship care plans, and palliative care. Patients and partners are invited to discuss their experiences around the session topics with the interventionist and ask questions about the information presented. However, patients and partners in this condition do not receive training in communication skills nor are they encouraged to disclose emotions or problem solve with each other. Following each session, the couples are encouraged to review the session handouts and utilize the information provided as appropriate.

An education intervention serves as an excellent control in this study since it involves both members of the couple and equates for time and attention given to couples. Analyses in our previous trials indicate that participants view educational interventions as highly credible [21, 22] and that we can successfully teach therapists how to administer specific interventions in a given treatment condition and maintain adherence while avoiding “bleed over” across conditions [33].

### Interventionist training and fidelity

Sessions are conducted by therapists with a master’s or doctoral degree in a mental health field. Therapists received initial training in the protocols which included background readings on key topics (e.g., common side effects of cancer treatment, principles of cognitive behavioral couple therapy, cultural considerations) and review of the intervention protocols. They then attended a training workshop which included review of readings and protocols and role plays of intervention delivery. They follow a detailed treatment manual which provides flexible guidelines for implementing the intervention protocols. All intervention sessions are audio-recorded. Supervision with the study investigators (L.S.P., K.R., T.J.S., K.S., F.J.K., D.H.B) occurs regularly and includes review of session audio recordings and supervisor-completed adherence forms, as well as discussions of intervention delivery and case issues. A random selection of at least 15% of sessions will be reviewed to assess intervention fidelity.

### Safety monitoring

Adverse events are assessed at each study visit. Given that patients in this study have advanced cancer, for this study protocol, the definition of an adverse event does not include worsening of any their medical condition as this is an anticipated occurrence. Responses are categorized according to the Common Terminology for Criteria for Adverse Events version 5.0. The research team will keep a log of the tracking the number, nature, and frequency of adverse events as part of each phase of the research plan. For any problem or event requiring prompt reporting to the IRB, within ten business days of the investigator becoming aware of the event, study personnel will send to the IRB a Safety Event submission in the Duke University IRB portal. Other adverse events (not related to the study) will be monitored and recorded and submitted to the IRB with the annual review. Severe adverse events are reported immediately (within 24 h).

### Data collection

Self-report measures are administered via REDCap, which may reduce error due to manual entry. Study staff routinely checks completed forms for quality assurance. Participants without computer access can complete paper and pencil versions of the consent forms and measures. After providing informed consent, patients and partners complete the baseline assessment (T1) which includes completion of self-report measures and a 10-min video-recorded couple conversation about a cancer-related topic of their choosing. After the third intervention session (approximately 4 weeks following T1), all participants complete the mid-treatment assessment (T2; self-report measures only). At the end of treatment (approximately 7 weeks following T1), they complete the T3 assessment which includes both self-report measures and the couple conversation. Self-report measures are administered again at 3-months post-treatment (T4). The timing of assessments will vary somewhat depending on the rate at which intervention sessions are completed, as the protocol allows the six sessions to be completed in as few as six or as many as 14 weeks. At each assessment, participants receive an email with a link to the REDCap survey. If participants do not complete the survey, an automated email reminder is sent every 5 days for up to 1 month; the study coordinator also calls participants to ensure that they have received the email link and can access the survey. All participants who are randomized to treatment are asked to complete follow-up assessments, regardless of whether they complete all six intervention sessions. Participants are paid a total of $150 for completing all four assessments (T1 = $30, T2 = $30, T3 = $40, T4 = $50).

Methods to protect the confidentiality of participant data include identifying participants only be a unique study number, maintaining electronic records using a dedicated database housed in an encrypted and password-protected file server, storing files in paper format in secure cabinets under lock and key, and limiting access to only those on the study team who require identifiable data.

### Outcome assessments

We collect the following data on enrolled patients through medical chart review: date of diagnosis and disease stage, dates and types of treatments and surgeries received for cancer, current treatment status, and current medication use. The data abstraction is performed by study staff and entered into our de-identified databases. This medical chart review is documented in the consent form.

#### Primary outcomes: relationship functioning

*Intimacy* is measured by the Miller Social Intimacy Scale (MSIS) [34]. The MSIS assesses intimacy experienced in relationships. Each of the MSIS’ 17 items is scored on a 10-point scale ranging from one (very rarely) to 10 (almost always). Miller and Lefcourt [34] demonstrated MSIS’ validity, and studies have confirmed its consistency (Cronbach alpha = 0.91) as well as its reliability (*r* = 0.96 over a 2-month interval).

*Relationship satisfaction* is assessed using the Couple Satisfaction Index-8 (CSI-8). This 8-item scale is an adaptation of the longer 32 version of the measure [35], created by the author of the original scale to address specific needs of treatment outcome research. The items are totaled for a range of 0–41 with higher scores represent higher levels of satisfaction. The original CSI has demonstrated strong convergent validity with other measures of relationship satisfaction, and excellent construct validity [35].

#### Secondary outcomes

*Psychological adjustment* is measured by scales assessing anxiety and depression [Hospital Anxiety and Depression Scale (HADS)] [36], positive and negative affect [Positive and Negative Affect Scale (PANAS)] [37], and a sense of meaning and peace in the context of serious illness [Life Completion subscale of the Quality of Life at the End of Life (QUAL-E)] [38, 39]. The HADS is a 14-item instrument that assesses anxiety and depression as two dimensions. This scale has been widely used to assess psychological distress in cancer patients [40]. The PANAS consists of two 10-item subscales, one measuring positive affect and the other negative affect [37]. Examples of items assessing positive affect are “excited,” “alert,” and “inspired” while descriptors for negative items are “guilty,” “irritable,” and “hostile.” Participants indicate how they have felt over the past week by rating each item on a scale from 1 (very slightly or not at all) to 5 (extremely). The seven item Life Completion subscale of the QUAL-E assesses individuals’ sense of meaning and peace, as well as the degree to which they have felt cared for and able to care for others. Its alpha coefficient is 0.80, and its reliability and validity have been confirmed by Steinhauser and colleagues [41, 42]. There are separate versions for patients and family members.

*Patient physical well-being* is measured by the Physical Well-being subscale of the Functional Assessment of Cancer Therapy (FACT) [43]. This subscale contains seven items scored from zero (not at all) to four (very much) assessing symptoms such as pain and nausea and their impact on patient quality of life. The FACT subscales have excellent psychometric properties and are widely used to assess quality of life among patients with cancer [44].

*Patient symptoms* are measured by the physical symptom subscale of the Condensed Memorial Symptom Assessment Scale (CMSAS) [45] which assesses the presence and bother of eleven common cancer-related symptoms (e.g., lack of energy, lack of appetite, drowsiness). For each symptom, patients report its presence (yes/no), and, if present, how much the symptom bothers them on a scale from 0 (not at all) to 4 (very much). Studies have found the CMSAS to capture information on quality of life and survival equivalent to that assessed by the original 32-item Memorial Symptom Assessment Scale Short-Form scale [45, 46].

*Patient health care use, advanced care plans, and advanced directives* are collected via both self-report and electronic medical record review. Patients are asked to report how many times they have gone to the emergency department and how many overnight hospital stays they have had in the past month (T1) or since the last assessment (T2-T4). They are also asked whether they have discussed advance care planning with their health care providers and whether they have completed an advance directive.

#### Mediators

*Self-reported communication*. Patients and partners complete the Protective Buffering Scale (PBS) [47] which assesses avoidance of discussing cancer-related concerns to protect the other person. Participants rate the extent to which they deny or hide their anger; worries; avoid disagreeing with their partner; give in more during arguments; act more positive than they feel; avoid talking about things; and withhold upsetting information. Each item is rated on a 1–5 scale, with higher values indicative of greater buffering. The PBS is a widely used measure of cancer-related communication, and has demonstrated strong psychometric properties [48].

*Objective indices of communication*. Objective indices of communication include use of the Linguistic Inquiry and Word Count (LIWC) [49] to identify first-person plural pronoun use (“we-talk”) during the 10-minute couple conversations. LIWC is one of the most extensively validated text analysis programs. It processes text word-by-word and yields a percentage of all words in the transcript that fall into a set of different linguistic and psychological categories. Analyses will focus on the percentage of use of first-person plural pronouns (“we,” “us,” “our”) as an implicit measure of communal coping. Greater use of “we-talk” among couples is indicative of a communal coping approach in which couples view a problem as a shared rather than individual problem [50, 51] In previous studies, “we-talk” by oneself and one’s partner has been associated with marital satisfaction, positive emotional behavior, effective problem solving, and decreased distress over time [52, 53].

The second objective index of communication involves observational coding of the couple conversations. The conversations will be coded using the Asymmetric Behavior Coding System (ABCS) to assess communication behavior and the Relational Affective Topography System (RATS) to assess emotional expression [54]. The systems are based on the Valence Affective Connection model [55] and delineate communication behavior and emotional expression into those that are positive or negative and those that promote togetherness or engagement with the partner [ABCS: positive approach (e.g., disclosure), negative approach (e.g., blame); RATS: positive joining (e.g., warmth), soft negative emotional expression (e.g., sadness)] and those that facilitate individuation or separation from the partner [ABCS: positive individuating (e.g., accommodation), negative individuating (e.g., avoidance); RATS: positive individuating (e.g., satisfaction), hard negative emotional expression (e.g., anger)]. For the RATS, coders will rate the extent to which they observe the emotions expressed on a scale of 0 (no emotion present) to 7 (high levels of emotion present). For the ABCS, coders will rate communication behavior on a scale of 1 (no behavior present) to 7 (high levels of behavior present). There will be separate coding teams for the two coding systems. Coders will be initially trained in the coding systems and then supervised on a weekly basis to maintain reliability.

#### Implementation-related process evaluation

The purpose of the process evaluation is to collect information to inform future efforts to implement CCST in clinical practice [56, 57]. To that end, the evaluation will address a series of questions informed by the RE-AIM (Reach, Efficacy, Adoption, Implementation, and Maintenance) evaluation framework (Table 3). The questions described in Table 3 will be addressed using the following methods:

**Table 3:** Process evaluation questions and data collection activities informed by RE-AIM

Element	Level	Method	Questions	Data Sources
**Reach**	Patient and partner	Quantitative	What percentage of patients approached agree to participate? Do patients who agree to participate differ systematically from those who decline participation? What reasons do non-participants cite for declining participation?	Recruitment rates Characteristics of those agreeing to vs. declining participation Patient and partner reports during recruitment process
**Adoption**	Setting	Qualitative	What are anticipated barriers and facilitators to providers and clinics adopting the intervention? What supports will need to be in place for providers and clinics to adopt the intervention?	Perceptions of oncology and palliative care providers and leadership
**Implementation**	Patient and partner	Mixed	What percentage of couples who agreed to participate in the intervention completed all intervention sessions? What did patients and partners like or not like about the intervention? What modifications would they suggest?	Completion rates Patient/partner responses on post-intervention survey
**Maintenance**	Setting	Qualitative	What resources will be needed to maintain the intervention long term? What adaptations will need to be made to integrate the intervention into routine care?	Perceptions of oncology and palliative care providers and leadership

*Study databases*: To evaluate Reach of the intervention, we will maintain a recruitment database with basic demographic and diagnostic information on every patient approached regarding participation in the intervention. Participants who decline participation will be asked to provide a reason for declining to help inform future efforts to improve Reach. In addition, we will track completion of all intervention sessions among participating couples to evaluate Implementation or the percentage of couples who complete all intervention sessions.

*Patient and partner surveys*: To collect information that could help to improve Implementation, both members of participating couples will complete post-intervention surveys including closed- and open-ended questions about what they liked and did not like about the intervention and what modifications they would recommend be made.

*Provider focus groups*: To collect information relevant to Adoption and Maintenance, after all couples have completed the intervention, we will conduct a focus group with oncology providers in the Duke Cancer Institute (DCI) breast, lung, GI, and GU clinics and with Duke palliative care providers. To inform future research on implementation of CCST in community (or non-academic) settings, we will conduct an additional focus group with community-based providers identified with the help of the DCI Community Advisory Council. We will solicit providers’ opinions regarding the intervention and anticipated barriers and facilitators to adopting and maintaining the intervention in practice (e.g., anticipated impact of screening and referring to CCST on their interactions with patients and clinic workflow).

*Leadership interviews*: To further inform Adoption and Maintenance, we will conduct post-intervention interviews with clinical and operations leaders of the DCI breast, lung, GI, GU programs, and the Duke palliative care program and with leaders from two community-based practices represented in our provider focus groups. Questions will focus on the possibility of and incentives for adopting CCST and types of support needed for adoption and maintenance of the intervention.

### Analyses

All the proposed primary and secondary analyses focus on the effect of the CCST (intervention) arm as compared to the Education (control) arm. We, therefore, plan to use the intent-to-treat assumption for all analyses; participants will be analyzed as part of the group to which they are randomized, regardless of intervention adherence. No interim analyses are planned.

*Missing data*. Our main analysis technique, multilevel linear models via maximum likelihood estimation, implicitly accommodates missingness when missingness is due either to treatment, to prior outcome, or to other baseline covariates included in the model [58]. Therefore, inferences will be valid even if we have differential dropout by treatment group. As a sensitivity analysis, but depending on the type and scope of missing data, we will also explore multiple imputation—via the SAS procedure PROC MI or the SAS macro IVEware (http://www.isr.umich.edu/src/smp/ive/)—as a strategy to use in conjunction with our primary analytic tools [59].

*Analysis of aims 1 and 2*. As described in the measures section, the MSIS and CSI-8 will be measured on all participants at baseline, post-treatment, and 3 months of follow up. A multilevel linear model will be used to estimate changes in intimacy and relationship satisfaction over time and test the primary hypothesis. Multilevel linear models are a flexible and powerful analytic tool appropriate for couples’ treatment studies because they account for the multiple sources of correlation arising from repeated measures nested within individuals nested within couples. As described by Atkins [60], the following model will be the basis for the primary analysis: *Y*_*tij*_*= β*_*0*_+ time*β_*1*_+ time*treatment*β_*2*_+ cancersite*β_*3*_+ cancerstage*β_*4*_*+ c*_*0j*_+ time*c_*1j*_*+ b*_*0ij*_*+ e*_*tij*_ [1], where *Y*_*ti*j_ is the outcome variable (e.g., CSI-8 or MSIS) for individual *i* of couple *j* at time point *t*. The random effects include a couples’ random intercept (*c*_*0i*_) and slope (*c*_*1i*_), the individual random intercept (*b*_*0ij*_), and the random residual error (*e*_*tij*_). The model assumes homoscedasticity and normality of all random effects.

The fixed effects include a common intercept [61] time and the interaction of treatment group and time. The stratification variables of patient sex and cancer type are also included. We will estimate the parameters in the model using the SAS procedure MIXED (SAS Version 9.2, Cary, NC); a significant estimate for the treatment by time interaction (*β*_*2*_) represents improvement in the intervention arm as compared to the control arm. A possible extension to the model would be to include an additional fixed effect for role, allowing patients and partners to have different intercepts, but common slopes over time. Similar multilevel models will be used for the individual functioning (HADS, PANAS, Life Completion) in Aim 2. Analyses of patient health/health care outcomes in Aim 2 will be conducted using a modified version of this model which includes patient data only.

*Analysis of aim 3*: This aim is to determine whether for couples receiving the intervention, improvements in individual and relationship functioning can be accounted for by improvements in their communication, including self-reports of protective buffering, observer ratings, and use of first-person plural pronouns (“we-talk”). We will assess (a) whether improvements in outcomes from baseline to post-intervention are accounted for by decreases in self-reported protective buffering from baseline to mid-treatment and (b) whether improvements in outcomes from baseline to 3-month follow up are accounted for by improvements in self-reported and objective indices of communication from baseline to post-intervention.

This aim can be addressed under the general framework of mediation. We propose to conduct this mediation analysis using the MacArthur approach, a modification of the traditional Baron and Kenny criteria, developed for use specifically in randomized clinical trials [62, 63]. The change in communication measures between baseline and mid-treatment and between baseline and post-treatment will be computed and these change scores will be included in the models to assess the mediation impact of change in communication. For mediation to occur, treatment must impact communication, and, in turn, communication must impact the outcomes at post-intervention or follow-up.

We will first fit a model to examine the correlation between change in protective buffering (baseline to mid-treatment) and treatment arm: *C*_*mid*_ = γ_0_ + γ_1_*treatment. In a second step, we will fit a model examining the relationship between communication at mid-treatment and outcomes at the post-treatment assessment: *Y*_*post*_*= β*_*0*_+ treatment*β_*1*_*+ C*_mid_*β_*2*_*+ C*_mid_* treatment *β_*3*_. Similar models will be used to examine changes in protective buffering, observed communication, and pronoun use from baseline to post-treatment, and outcomes collected at the three-month follow up assessment. Improvements in communication will be considered to partially account for (mediate) the impact of the intervention on the outcomes if there is evidence that γ_1_ is not equal to 0, and either *β*_2_ or *β*_2_ are not equal to zero. The degree of mediation can be computed by multiplying the coefficients above, while *p*-values can be computed by the methods listed in Preacher [64].

*Analyses of implementation-related data*: Chi-square tests, and *Z* or *t*-tests will be used to compare characteristics of patients who accepted vs. declined the intervention. Descriptive quantitative results will be reported in percentages. Qualitative data from open-ended survey questions, interviews, and focus groups will be managed and evaluated in a systematic format [65] and will be used to assess issues related to delivery and implementation of the CCST intervention for a future implementation trial.

### Sample size and power considerations

The sample size estimate for this study is based on the first primary hypothesis that patients and partners in the intervention arm will have improved relationship functioning as measured by the Miller Social Intimacy Scale (MSIS). Power and sample size estimates are generated by simulation. In the simulation, model [1] was fit to each dataset and the *p*-value for the treatment effect (b2) was saved. The number of times the *p*-value is < 0.05 divided by the total number of simulated datasets estimates the power of the test for a type-I error of 0.05. All power calculations below are based upon 1000 simulated datasets.

Data from our previous study [27] was used to generate estimates for the variance components of the random effects. Combining estimates on MSIS from two previous studies (mean = 8.22, SD = 1.12, within-couple correlation rho of 0.42), power analysis conducted in simulation program PASS 2021, yield a sample *n* = 80 couples in each group to attain 90% power to detect an adjusted Cohen’s *d* around 0.33 (a small to moderate effect size), at 5% level of significance, which corresponds to approximately 6% increase in the treatment group mean from the control group mean. Given that patients in the study have advanced disease, we conservatively estimate a 30% attrition rate by the 3-month follow up; therefore, we plan to enroll and randomize 230 couples (115 in each arm).

### Ethical aspects

The trial has been approved by the Institutional Review Board (IRB) at Duke University Medical Center (Protocol # Pro00103232). All members of the study team have been trained in principles of ethical conduct of human subjects’ research and in compliance with study procedures. Potential participants are informed that their decision regarding whether or not to participate in the study will not affect their receipt of healthcare. Study participants are informed that they can discontinue their participation in the study at any time; this is indicated in the consent forms. Given the minimal risk of this study, it was determined that a Data and Safety Monitoring Board is not necessary. All study activities, including ethical conduct, regulatory compliance, and recruitment and retention, are reviewed annually by the Duke IRB and Cancer Protocol Committee. Additional oversight is provided by the Duke Psychiatry Department Clinical Research Unit which conducts quarterly audits of compliance with regulatory procedures, and the study sponsor which monitors study progress annually. The investigator team meets weekly to discuss recruitment, retention, data collection, and treatment fidelity. Adverse events are reviewed annually; issues with intervention delivery and other unintended consequences of either intervention are discussed during regular supervision meetings. Significant protocol modifications will be approved by the IRB and reported to relevant parties (e.g., clinicaltrials.gov) in a timely fashion. Adverse events are reported immediately to the Principal Investigator, tracked, and responded to according to regulatory guidelines. In the rare event that a participant is experiencing severe psychological or relational distress that cannot be managed within the context of the intervention sessions, the principal investigator will recommend that the couple discontinue in the study and will refer them for appropriate care. Study interventions can be modified in minor ways in response to participant needs (e.g., modifying the order in which educational topics are presented); this will be determined by the Principal Investigator in consultation with the study therapist. As harm from this type of study is rare, there are no provisions in place for ancillary, post-trial, or compensation for study-related harms.

## Discussion

To our knowledge, this study is the first large-scale RCT to test the efficacy of a couple communication skills training intervention in advanced cancer. In our approach, we assess and intervene on communication skills and topics that pose challenges for both patient and partner. We also screen participants for inclusion based on communication difficulties to ensure that the intervention is targeted to those at risk for poor outcomes and use an attention control condition which controls for non-specific therapeutic effects including the time couples spend together in the sessions and attention from the therapist. If CCST is found to be effective, this study will advance clinical practice through the identification of a screening tool to detect couples who are having difficulty addressing cancer-related issues and a brief, scalable intervention that can assist them. The study also includes an implementation-related process evaluation of the intervention with the goal of expediting translation of the intervention into practice.

Our trial is also novel in that it will systematically evaluate mechanisms by which the CCST intervention produces improvements in patient and partner relationship and psychological adaptation and patient health. Specifically, we will examine the role of communication as a potential mediator using novel indices of communication including observer ratings of communication skills, linguistic analysis of first-person pronoun use (“we-talk”), and self-reports of protective buffering. By understanding the mechanisms of the CCST intervention (e.g., how it works), we can optimize improvements in outcomes and accelerate translational research.

Another distinctive feature of the study is that our secondary aims include examining the impact of the CCST intervention on patient health outcomes relevant to palliative care, including advance care planning discussions and the completion of advance directives. Advance care planning is the process that supports patients in understanding and sharing their personal values, life goals, and preferences regarding future medical care with their family and health care providers [66]. Given the central role that family members play in advance care planning [67], facilitating patient-partner discussions about their hopes, fears, and treatment preferences may help them come to decisions together about current and future medical care. Advance care planning and advance directives benefit both patients and partners, helping ensure patients receive care consistent with their preferences, providing guidance to the family and reducing their decisional burden if they must act as the patient’s surrogate [67], reducing hospitalizations and aggressive treatments at the end of life [68], and decreasing distress for both patients and family. Despite widespread efforts among health care providers to encourage advance care planning, the majority of patients with advanced cancer do not have an advance care planning conversation before death [69]. By preparing patients and partners to engage in high-quality discussions about medical decisions, the CCST intervention may be effective in increasing advance care planning discussions and/or directives and improving good end-of-life care.

Lastly, our videoconference format is an accessible delivery approach that can aid in assessment and dissemination of treatment while also supporting family-centered palliative care research and practice. In palliative care, family members are considered part of the unit of care, yet as dyadic entities, they are underrepresented in research. If found to be effective, the major elements of the CCST intervention could be integrated into routine palliative care. It could also be applied to the larger population of patients with other serious health conditions, and adapted for other types of dyads (e.g., to make it available to patients who do not have an intimate partner but have other individuals involved in their care such as an adult child or friend).

There are several limitations to the study. First, participation in the study is restricted to dyads who are married or in a committed intimate relationship. This approach is based on prior research indicating that the patient-partner relationship and their communication in particular can play a critical role in helping patients with cancer adapt to their illness [26, 29]. However, the findings from the study may not generalize to patients with cancer who are not married/partnered and their informal caregivers (e.g., adult children or friends). As noted above, future studies should examine how best to adapt the CCST intervention to meet the needs of other types of dyads. Second, the CCST intervention focuses primarily on communication behaviors, for example encouraging positive behaviors such as disclosure and discouraging negative behaviors such as blame or avoidance. Findings from analyses of observational data may identify other aspects of the communication process such as emotional expression that are important to address within the intervention. Third, the videoconference format may challenging for participants who lack reliable internet access at home (e.g., rural communities), or who do not have a device to use for videoconference or are uncomfortable using the technology. To mitigate these challenges, as we have in previous studies we are offering tablet computers with internet access and instructions in their use to those who need them, and we offer telephone sessions as an alternative in the event videoconferencing is not possible. Also, as the part of the process evaluation, we will be explicitly evaluating reach and barriers to participation so that we can adapt in the future. Finally, the study is being conducted at a single academic medical center in the Southeast. Thus, participants may not be fully representative of the national population or of patients who seek care at community hospitals.

Despite these limitations, this study has the potential to advance clinical care through the development and evaluation of an evidence-based intervention to improve the adjustment of patients with cancer and their caregiving partners. The findings of this trial will be disseminated through the study’s entry on ClinicalTrials.gov, publication in peer-reviewed journals, and presentation of findings at scientific conferences. A summary of the findings will also be shared with study participants at the end of the trial.

In conclusion, this study is among the first RCTs to test the efficacy of an intervention to improve the way couples communicate about important concerns related to advanced cancer. By teaching both patient and partner skills for disclosing thoughts and feelings about cancer, along with communication strategies for accepting and affirming the other person’s feelings and perspectives and making decisions together as a couple, we aim to improve their individual and relationship adjustment as well as patient health and health care outcomes. Our intervention represents a potentially sustainable approach to help patients and their partners address the formidable challenges posed by advanced cancer that affect them as individuals and as a couple. It is innovative in its application of a brief, targeted, and potentially scalable intervention to couples who are most at risk for poor outcomes in advanced cancer.

## Supplementary Information

**Additional file 1:.** SPIRIT Checklist. Detailed checklist of items in the SPIRIT Checklist.

**Additional file 2:.** Consent form. Model consent form.

## Data Availability

Study data and relevant materials from the trial described in this manuscript will be retained and archived by the primary study site for a minimum of 3 years after study completion as per NIH policy on record retention. There are no plans for publicly sharing the trial data. No material (biological specimens) are collected as part of this trial.

## Abbreviations

ABCS: Asymmetric Behavior Coding System
CMSAS: Condensed Memorial Symptom Assessment Scale
CCST: Couple Communication Skills Training
CSI: Couple Satisfaction Inventory
DCI: Duke Cancer Institute
FACT: Functional Assessment of Cancer Therapies
GI: Gastrointestinal
GU: Genitourinary
HIPAA: Health Insurance Portability and Accountability Act
HADS: Hospital Anxiety and Depression Scale
IRB: Institutional Review Board
LIWC: Linguistic Inquiry and Word Count
MSIS: Miller Social Intimacy Scale
PANAS: Positive and Negative Affect Scale
PBS: Protective Buffering Scale
QUAL-E: Quality of Life at End of Life
RCT: Randomized controlled trial
RE-AIM: Reach, Efficacy, Adoption, Implementation, and Maintenance
RATS: Relational Affective Topography System
