# Supplementary material for: Evaluating a couple communication skills training (CCST) intervention for advanced cancer: study protocol for a randomized controlled trial
Source: Trials. 2022 Aug 26;23:712. doi: 10.1186/s13063-022-06656-4 (PMC9419413; doi:10.1186/s13063-022-06656-4)
Supplement: Supplementary file 2 — Additional file 2:. Consent form. Model consent form. [file 13063_2022_6656_MOESM2_ESM.docx]

Coping Together: Couple-based interventions for cancer

We are asking you to take part in this research study because you have been or your partner has been diagnosed with breast, gastrointestinal, genitourinary or lung cancer.

Research studies include only people who choose to take part. Please read this consent form carefully and take your time making your decision. Feel free to ask questions is there are any words or information that you do not clearly understand. We suggest that you talk with your family and friends before you decide to take part in this research study. The nature of the study, risks, inconveniences, discomforts, and other important information about the study are listed below.

Please tell the study doctor or study staff if you are taking part in another research study.

CONCISE SUMMARY

The purpose of this study is to see whether couple-based programs are helpful for couples in which one partner has cancer. You and your spouse/partner will have six, 60-minute video-conference sessions which will be scheduled at your convenience. We will loan you a tablet computer (iPad) to use for video-conferencing and train you in its use. You and your spouse/partner will complete four assessments – one before starting the sessions, one after the third session, one after the sixth session, and one after 3 months. Each assessment will include surveys, which you will complete separately from your spouse/partner. The first and third assessment will also include a video recorded10minute conversation between you and your spouse/partner about a cancer-related topic of your choosing. For most people, it will take 4 to 6 months to complete this study.

The greatest risk of being in this study is that some of the topics covered in the sessions or in the surveys will make your or your partner feel some increased distress.

A grant from the National Institutes of Health (NIH) will sponsor this study. Portions of Dr. Laura Porter's and his/her research team’s salaries will be paid by this grant.

**WHO WILL BE MY DOCTOR ON THIS STUDY?**

If you decide to participate, your medical care will continue as usual.

**WHY IS THIS STUDY BEING DONE?**

We are doing this research to see whether couple-based programs are helpful for couples in which one partner has cancer. We are looking at the usefulness of two programs: (1) Couple Communication and Support, and (2) Healthy Lifestyle Information.

**HOW MANY PEOPLE WILL TAKE PART IN THIS STUDY?**

Approximately 460 people (230 couples) will take part in this study at Duke.

Coping Together: Couple-based interventions for cancer

**WHAT IS INVOLVED IN THE STUDY?**

If you take part in this study, you will be asked to sign and date this consent form.

You will complete online surveys separately from your partner and participate in a six-session program with your partner over video-conference. You each will fill out your own consent form. You will both be considered research participants in this study.

If you agree to be in this study, we will ask you to do the following things:

# Study Programs

You will be "randomized" into one of two research study programs. This means that you are put into a program by chance, like flipping a coin. A computer program will place you and your partner, as a couple, in one of the research study programs. Neither you nor your doctor can choose the program you will be in. You will have an equal chance of being placed in either program. The first program, Couple Communication and Support (CCS) focuses on ways you and your partner can support each other and communicate about issues and concerns related to cancer. The second program, Healthy Lifestyle Information (HLI), provides you and your partner with information and resources on topics relevant to maintaining your health during cancer treatment (such as sleep, nutrition, and exercise).

Both programs consist of six, 60-minute private video-conference sessions with a facilitator. You will take part in these sessions along with your partner. We will schedule these sessions a time that is good for you. They will be spaced about a week apart. In the case that sessions need to be rescheduled or scheduled further apart, we ask that couples complete these six sessions within twelve weeks.

All sessions will be audiotaped for quality control. **By consenting today, you agree to let us audio record the sessions.** Members of the study team at Duke University may review the recordings for quality control or training purposes.

**Use of Duke loaned devices:**

If you are loaned a Duke iPad for use during this study and you use it for non-study related reasons, this could add your personal information onto the device and potentially result in it being sent to unauthorized persons. The device will be preset with security settings. Please do not alter these during the course of the study. When you return the device at the end of the study, the device will be cleaned to remove any of your personal information. If the device is lost or stolen during the course of the study, please contact the study team immediately.

Assessments - surveys and couple conversations:

Surveys: We are asking you to fill out surveys for this research study which can be done either through the web (using a link we give only to you) or through US Mail. These surveys will ask things about you Coping Together: Couple-based interventions for cancer

(such as your race/ethnicity, education, and work) and your relationship. There are other surveys that ask about your physical health along with any medical problems and emotional health, such as about depression and anxiety symptoms, intimacy, relationship quality, and communication. You do not have to answer any question that you do not want to. You will spend about 30 minutes filling out the surveys. We will ask you to fill out these surveys 4 times during this research study. We will ask you to do all surveys **separately** from your partner. We will not share your answers to the questions with your partner. You do not have to answer any questions that make you uneasy. Whether or not you answer any question will not affect your medical care. To protect your privacy, only the research team will see your answers to the survey questions.

Couple conversations: Once consented we will ask you to have a video recorded conversation with your partner to share your thoughts and feelings about a topic of importance to both of you related to the cancer, treatment, and/or recovery. The conversation will last 10 minutes (or less, if you didn’t want to talk that long).When the interventions are completed we will ask you to have another conversation with your partner that will be video recorded. Allrecordings will be destroyed within five years of study completion.

# Medical Records Review

If you are the patient, we will ask you if it is ok to look in your medical records for information about your cancer. This will include your cancer diagnosis, dates and types of treatments and surgeries, and other aspects of your medical history, such as the medicines you take. We will add this information to our private research files. We will only collect information that is needed for our study and any information we put into our study files from your medical records will not have your name or medical record number with it.

If you are the partner (not the patient with a cancer history), we will not go into your medical records.

**HOW LONG WILL I BE IN THIS STUDY?**

For most people, it will take about four to six months to complete the study.

Taking part in this study is your choice. You can agree to be in the study now and change your mind later.

- If you wish to stop, please tell us right away.
- Leaving this study early will not stop you from getting regular medical care

**WHAT ARE THE RISKS OF THE STUDY?**

There are no physical risks or side effects expected to happen in this study. There is a possible risk of loss of privacy. We will make every effort to keep your information private, but this cannot be guaranteed.

Coping Together: Couple-based interventions for cancer

You may get tired or bored when we are asking you questions or you are filling out surveys. Also, some of the questions we will ask you, and topics that may come up as part of this study may be sensitive.. It is possible that you and your partner may feel some added distress talking about these issues while taking part in this study. You do not have to answer any question you do not want to answer and you may take a break at any time during the study. You may also stop being in this study at any time.

We do not expect that taking part in this study should cause added distress or relationship problems for you and/or your partner. But if you have relationship problems during the course of this study, we will do all we can to help you and your partner deal with these issues. If you feel that you should not stay in the study because of these problems or any distress, we will suggest that you leave the study. We will offer you referrals for resources including a marital counselor or social worker, if needed. Whether or not you are having relationship problems, you may stop taking part in this study at any time.

**ARE THERE BENEFITS TO TAKING PART IN THE STUDY?**

Taking part in the study may or may not help you directly. You and your partner may learn helpful information through taking part which could be of use to you. You may find it helpful to talk about aspects of your relationship or your health with a counselor. You or your partner may see improvements in aspects of your emotional and relationship well-being but there are no guarantees of this. By taking part, you could help us find better ways of supporting cancer patients and their spouses/partners. This may help cancer patients and their partners in the future.

**WILL MY INFORMATION BE KEPT CONFIDENTIAL?**

We will do our best to make sure that the personal information in your medical record will be kept private. However, we cannot guarantee total privacy. Your personal information may be given out if required by law. If information from this study is published or presented at scientific meetings, your name and other personal information will not be used.

The Department of Health and Human Services (HHS) has issued a Certificate of Confidentiality to further protect your privacy. With this Certificate, the investigators may not disclose research information that may identify you in any Federal, State, or local civil, criminal, administrative, legislative, or other proceedings, unless you have consented for this use. Research information protected by this Certificate cannot be disclosed to anyone else who is not connected with the research unless:

1. there is a law that requires disclosure (such as to report child abuse or communicable diseases but not for legal proceedings);
2. you have consented to the disclosure, including for your medical treatment; or
3. the research information is used for other scientific research, as allowed by federal regulations protecting research subjects.

Coping Together: Couple-based interventions for cancer

Disclosure is required, however, for audit or program evaluation requested by the agency that is funding this project or for information that is required by the Food and Drug Administration (FDA).

You should understand that a Confidentiality Certificate does not prevent you or a member of your family from voluntarily releasing information about yourself or your involvement in this research. If you want your research information released to an insurer, medical care provider, or any other person not connected with the research, you must provide consent to allow the researchers to release it. This means that you and your family must also actively protect your own privacy.

Finally, you should understand that the investigator is not prevented from taking steps, including reporting to authorities, to prevent serious harm to yourself or others.

Organizations that may look at and/or copy your medical records for research, quality assurance, and data analysis include:

 The National Cancer Institute (NCI) and other government agencies, like the Food and Drug

Administration (FDA), involved in keeping research safe for people

A description of this study will be available on http://www.ClinicalTrials.gov, as required by U.S. Law. This website will not include information that can identify you. At most, the website will include a summary of the results. You can search this website at any time.

Some people or groups who receive your health information might not have to follow the same privacy rules. Once your information is shared outside of Duke, we cannot guarantee that it will remain private. If you decide to share private information with anyone not involved in the study, the federal law designed to protect your health information privacy may no longer apply to the information you have shared. Other laws may or may not protect sharing of private health information.

**WHAT ARE THE COSTS TO YOU?**

There are no costs to you for taking part in this study. No visits to Duke Health are needed as part of this study.

**WHAT ABOUT COMPENSATION?**

Those completing assessments (surveys and couple conversations) will be reimbursed as follows, in the form of debit cards:

Coping Together: Couple-based interventions for cancer

- $30 each for completing the first assessment ($60 per couple)
- $30 each for completing the second assessment ($60 per couple)
- $40 each for completing the third assessment ($80 per couple)
- $50 for completing the fourth assessment ($100 per couple)

We will send the debit cards through the US Mail upon receipt of the participant’s survey. Participants who complete all four surveys for this study will receive a total of $150 ($300 per couple).

**WHAT ABOUT MY RIGHTS TO DECLINE PARTICIPATION OR WITHDRAW FROM THE STUDY?**

You may choose not to be in the study. If you agree to be in the study, you may withdraw from the study at any time. If you withdraw from the study, no new data about you will be collected for study purposes other than data needed to keep track of your withdrawal. All data that have already been collected for study purposes will be sent to the study sponsor.

Your decision not to participate or to withdraw from the study will not involve any penalty or loss of benefits to which you are entitled, and will not affect your access to health care at Duke. If you do decide to withdraw, we ask that you contact study staff to let them know you want to withdraw.

We will tell you about new information that may affect your health, welfare, or willingness to stay in this study.

A description of this clinical trial will be available on <https://clinicaltrials.gov/> as required by U.S. Law. This Web site will not include information that can identify you. At most, the Web site will include a summary of the results. You can search this Web site at any time.

**WHOM DO I CALL IF I HAVE QUESTIONS OR PROBLEMS?**

For questions about the study or a research-related injury, or if you have problems, concerns, questions or suggestions about the research, contact Dr. Laura Porter at 919-416-3436 during regular business hours and at (xxx-xxx-xxxx) after hours and on weekends and holidays.

For questions about your rights as a research participant, or to discuss problems, concerns or suggestions related to the research, or to obtain information or offer input about the research, contact the Duke University Health System Institutional Review Board (IRB) Office at (919) 668-5111.

**STATEMENT OF CONSENT**

"The purpose of this study, procedures to be followed, risks and benefits have been explained to me. I have been allowed to ask questions, and my questions have been answered to my satisfaction. I have been told whom to contact if I have questions, to discuss problems, concerns, or suggestions related to the research, or to obtain information or offer input about the research. I have read this consent form and

Coping Together: Couple-based interventions for cancer

agree to be in this study, with the understanding that I may withdraw at any time. I have been told that I will be given a signed and dated copy of this consent form."

__________________________________________ ___________ ____________

Signature of Subject Date Time

__________________________________________ ___________ ____________

Signature of Person Obtaining Consent Date Time

***(Optional)***

**____________________________________ _____________________** ____________

| **Signature of Principal Investigator**  ***(If applicable, add or substitute any of the following:)*** | **Date Time** |
| --- | --- |
| __________________________________________ | ___________ ____________ |
| Signature of Legal Representative  __________________________________________ Relationship to Subject | Date Time |

*
